# MicroRNAs as Diagnostic and Prognostic Biomarkers in Melanoma and Non-Melanoma Skin Cancers: An Updated Review

**DOI:** 10.3390/diagnostics16010051

**Published:** 2025-12-23

**Authors:** Alexandra Oiegar, Adrian Bogdan Tigu, Adrian Baican, Elisabeta Candrea, Mircea Negrutiu, Sorina Danescu

**Affiliations:** 1 Department of Dermatology, “Iuliu Hatieganu” University of Medicine and Pharmacy, 400012 Cluj-Napoca, Romania; alexandra.oiegar@elearn.umfcluj.ro (A.O.); negrutiu.mircea.ionut@elearn.umfcluj.ro (M.N.); 2 Personalized Medicine and Rare Diseases Department, MEDFUTURE—Institute for Biomedical Research, “Iuliu Hațieganu” University of Medicine and Pharmacy, 400012 Cluj-Napoca, Romania

**Keywords:** microRNA, melanoma, non-melanoma skin cancers, diagnostic, biomarkers

## Abstract

MicroRNAs (miRNAs) have emerged as critical post-transcriptional regulators in melanoma and non-melanoma skin cancers (NMSCs), yet their full biological and clinical significance remains incompletely defined. This review synthesizes current evidence on miRNA dysregulation across basal cell carcinoma (BCC), cutaneous squamous cell carcinoma (cSCC), Merkel cell carcinoma (MCC), and melanoma, emphasizing their diagnostic, prognostic, and therapeutic relevance. In BCC, distinct miRNA expression signatures differentiate tumor tissue from normal skin and correlate with histopathological subtypes. miR-383-5p, miR-4705, miR-145-5p, and miR-18a show strong diagnostic potential, while downregulation of miR-34a is consistently associated with greater tumor aggressiveness. Subtype-specific profiles further delineate superficial versus infiltrative lesions, highlighting miRNAs as markers of tumor behavior. cSCC similarly demonstrates characteristic miRNA alterations. miR-31 is markedly upregulated during the transition from actinic keratosis to invasive carcinoma, whereas high miR-205 and low miR-203 levels correlate with poor and favorable prognosis, respectively. Regarding MCC, many miRNAs such as miR-375 and miR-182 may present a clinical value for potential biomarkers, as they are upregulated in MCC. Merkel cell carcinoma has also been linked with Merkel cell polyomavirus (MCPyV). Melanoma exhibits a complex miRNA landscape, including oncogenic miR-18a-5p and miR-146a, and tumor-suppressive miR-128-3p. Several miRNAs correlate with metastatic potential, BRAF mutation status, and therapeutic resistance, particularly miR-181a/b, underscoring their potential as predictive biomarkers. Overall, current evidence supports miRNAs as promising diagnostic, prognostic, and predictive biomarkers in cutaneous oncology. Standardized methodologies and large-scale validation remain essential for their integration into routine clinical practice.

## 1. Introduction—The Role of miRNA in Skin Cancers

The skin functions as a dynamic immune organ in which the interplay between environmental damage, DNA repair mechanisms, and immune surveillance ultimately shapes the risk of malignant transformation [1,2]. Skin cancers currently represent the most common group of malignancies in Caucasian populations [3]. They are broadly categorized as melanoma and non-melanoma skin cancers (NMSCs). The NMSC group includes basal cell carcinoma (BCC), cutaneous squamous cell carcinoma (cSCC), basosquamous carcinoma, Merkel cell carcinoma (MCC), Bowen’s disease (BD), and actinic keratosis (AK). Each entity presents distinct biological features, etiological factors, and prognostic implications. Among these, BCC, cSCC, and MCC are particularly important due to their potential to invade deeper tissues and metastasize [4]. Melanoma, on the other hand, remains the most aggressive form of skin cancer and is currently ranked as the fifth most common cancer in both men and women [5,6].

MicroRNAs (miRNAs) are short, non-coding RNA molecules (typically 17–25 nucleotides in length) that play a central role in the post-transcriptional regulation of gene expression [1]. First identified in 1993, they represented a major paradigm shift in molecular biology. MiRNA biogenesis begins with the transcription of primary miRNAs (pri-miRNAs) from specific DNA (deoxyribonucleic acid) transcripts, which are subsequently processed into precursor miRNAs (pre-miRNAs) and ultimately into the mature miRNA species [7].

MiRNAs play a critical regulatory role in the pathogenesis and progression of skin cancers, including cSCC, BCC, MCC, BD, AK and melanoma. Being small RNA molecules, miRNAs can modulate gene expression and determine prost-transcriptional modulation, which influences cellular processes such as proliferation, apoptosis, differentiation, angiogenesis, immune responses, and epithelial-to-mesenchymal transition (EMT) [3,8].

In skin cancers and in oncology in general, miRNAs can be targeted by different therapeutic agents that could inhibit or stimulate miRNA expression; however, the off-target effect, pleiotropy, the impact on non-tumor cells, and tumor heterogeneity still need to be considered, as currently the therapeutic use of miRNAs is limited and lacks constant evidence of efficacy. Each miRNA can regulate multiple mRNAs and pathways, and each pathway is influenced by multiple miRNAs at the same time, making their targeting challenging [7,9,10,11].

Together with DNA methylation and histone modifications, miRNAs are responsible for the epigenetic changes induced at the DNA level, acting either as oncogenic or onco-suppressor factors. Thus, miRNAs can be investigated as diagnostic and prognostic biomarkers due to their stability and detectability in tissues and biological fluids, offering insights that may help in staging the disease and predicting therapeutic outcomes [5,12,13,14].

The aim of this review is to synthesize the up-to-date scientific evidence on the contribution of miRNAs to the diagnosis, pathogenesis, and prognostic assessment of the most common non-melanoma skin cancers (NMSCs) and melanoma skin cancers (MSCs). By focusing on the most recent and methodologically robust studies in the literature, this work intends to delineate how specific miRNA signatures influence tumor initiation and progression, how they can help discriminate between distinct tumor subtypes, and how they may function as biomarkers for disease course and clinical outcomes.

## 2. Non-Melanoma Skin Cancers

Basal cell carcinoma (BCC) is the most frequent skin malignancy and arises from cells of the basal layer of the epidermis and adnexal structures [15]. Although BCC is associated with very low mortality, it can generate substantial morbidity due to its capacity for local tissue destruction [16]. Despite being locally invasive and capable of causing marked damage to surrounding structures, BCC generally grows slowly and metastasizes only rarely. The vast majority of lesions occur on chronically sun-exposed areas, with the head and neck region being the most typical location [15].

Exposure to ultraviolet (UV) radiation remains the principal risk factor for BCC, particularly in individuals with Fitzpatrick skin phototypes I–II, light-colored eyes, and fair hair (blonde or red), often accompanied by freckles. Additional risk factors include chronic immunosuppression, a positive family history of skin cancer, severe childhood sunburns, indoor tanning, prior exposure to ionizing radiation, and contact with carcinogenic substances such as arsenic [16].

Basal cell carcinoma exhibits diverse clinical and histopathological characteristics and can be classified into several subtypes. The nodular variant is the most frequent, while other recognized forms include the micronodular, superficial, morpheaform, and infiltrative subtypes. However, many BCCs show overlapping features from more than one category, making precise subclassification challenging in a substantial proportion of cases [15].

The molecular basis of BCC reflects a complex interaction between genetic predisposition and acquired somatic mutations. Individuals can present a genetic predisposition to BCC onset through genetic syndromes, germline SNP variants, and heritable genetic features. Genetic syndromes like Gorlin syndrome, Bazex–Dupre–Christol syndrome and Rombo syndrome are associated with inherited susceptibility to BCC. Oncogenic signal transduction pathways that are implicated in the molecular pathogenesis of BCC are Sonic Hedgehog pathway (Shh) and the mitogen-activated protein kinase/extracellular signal-regulated kinase (MAPK/ERK) cascade. The polymorphism rs7837822 in the TP53 gene presents a strong association with BCC. P53 is a tumor suppressor that enhances the function of protein p21, which is an inhibitor of cell cycle regulator cyclin D kinase. P53 can also induce apoptosis, via BCL2 inhibition, which is an anti-apoptotic protein, and suppress the HH pathway protein SMO. The NOTCH signaling pathway plays an important role in the process of differentiation and proliferation of keratinocyte. Dysregulations of this pathway can be associated with skin abnormalities and cutaneous tumors. A study showed that NOTCH signaling in human nodular BCCs is depressed, and when treated with a NOTCH signaling peptide, increased apoptosis of BCC cells was observed. The NOTCH pathway is involved in the regulation of epidermal development through many processes. NOTCH signaling suppresses keratinocyte proliferation through two mechanisms: by inhibiting p63, a transcription factor that drives epidermal growth, and by inducing CDKN1A, a cell cycle inhibitor [17].

Cutaneous squamous cell carcinoma (cSCC) arises from keratinocytes and represents the second most common form of NMSC, with an incidence that continues to rise. Established risk factors include chronic UV exposure, immunosuppression, male sex, advanced age, and a prior history of cSCC. This tumor type may progress rapidly and accounts for a substantial proportion of NMSC-related mortality. Regional lymph node metastasis develops in approximately 5% of patients, and distant organ involvement can also occur [2,18].

Bowen’s disease (BD) is classified as an in situ squamous cell carcinoma (SCC), while actinic keratosis (AK) is considered a precancerous lesion that can progress to invasive SCC. Although both are strongly linked to SCC development, they display distinct histopathological features [18].

Similarly to cutaneous basal cell carcinoma, extrinsic factors involved in the development of cutaneous squamous cell carcinoma, together with the accumulation of genetic alterations, can lead to the formation of premalignant and malignant lesions, particularly when the internal defense mechanisms are impaired. DNA mutation can occur due to exogen factors, such as UV light, chemicals, or ionizing radiation and also due to endogen factors, like mitotic errors, errors in DNA repair, genome editing, and reactive oxygen species. Among human cancers, cSCC presents the greatest tumor mutation burden, with over 50 mutations per megabase of DNA. The genes most frequently involved in the molecular pathogenesis of cSCC are TP53, NOTCH1, NOTCH2, and FAT1. P53 is the most frequently mutated gene across human cancers, playing an important role also in cSCC development. In cutaneous squamous cell carcinoma, the p53 gene encodes a 53 kDa phosphoprotein that regulates cell cycle arrest, senescence, DNA repair, and apoptosis as a response to cellular stress. p53 activity can be disrupted by inherited or acquired TP53 mutations or indirectly through alterations in its signaling pathway. Loss-of-function mutations in NOTCH1–3 are common in cutaneous squamous cell carcinoma (cSCC) and sun-damaged skin, even without histologic dysplasia. NOTCH1–3 encode Notch proteins that regulate keratinocyte cell fate, self-renewal capacity, lineage commitment, proliferation, and survival [19].

Merkel cell carcinoma (MCC) is a rare but highly aggressive cutaneous malignancy, also referred to as neuroendocrine carcinoma of the skin, Toker tumor, or trabecular carcinoma. Merkel cells, located in the epidermis, function as mechanoreceptors and are derived from the epidermal lineage. They are classified as neuroendocrine cells due to their ability to produce small amine and polypeptide hormones. MCC arises from the uncontrolled proliferation of skin cells exhibiting Merkel cell-like characteristics. Owing to its aggressive behavior, effective management typically requires a combination of surgical excision, radiotherapy, and chemotherapy [20]. The median age at diagnosis for Merkel cell carcinoma (MCC) is approximately 75 years, with only around 12% of cases occurring in individuals younger than 60. MCC most commonly develops on sun-exposed areas, including the head, neck, and extremities, although it can also arise in less typical locations such as the buttocks, oral mucosa, penis, and vulva. Ultraviolet (UV) radiation is a primary etiological factor, while additional contributors include immunodeficiency, fair skin, advanced age (reflecting immune senescence), prior malignancies, and chronic inflammatory conditions [21].

## 3. Melanoma

Melanoma incidence has been increasing worldwide over the past decades, and it remains one of the most aggressive forms of skin cancer. Early detection significantly improves outcomes, with survival rates approaching 94% [22]. Melanoma differs from non-melanoma skin cancers in its ability to metastasize locally, to regional lymph nodes, and to distant organs. The risk of metastatic spread correlates with the tumor’s depth of invasion and the presence of ulceration in the primary lesion [22].

Melanoma develops from the malignant transformation of melanocytes, the cells responsible for producing melanin, a pigment that provides protection against ultraviolet radiation [23]. Melanoma typically presents as an irregularly pigmented lesion exhibiting multiple colors and may be associated with symptoms such as pruritus, bleeding, ulceration, or the presence of satellite lesions. When an atypical lesion is suspected, an excisional skin biopsy should be performed to establish the diagnosis. Management generally involves wide local excision; removal of any satellite lesions if present; and, in advanced cases, consideration of adjuvant therapies based on tumor stage [22].

Factors that increase the risk of developing melanoma include a family history of melanoma, fair skin (Fitzpatrick types I–II), sun exposure or tanning bed use, having numerous benign or atypical nevi, and immunosuppressive conditions [22].

Histological subtypes include superficial spreading melanoma, nodular melanoma, lentigo malignant melanoma, acral lentiginous melanoma [22].

Melanoma progression is influenced by multiple factors, particularly genetic alterations affecting oncogenes and tumor suppressor genes. Key genes implicated include cyclin-dependent kinase inhibitor 2A (CDKN2A), which encodes the cell cycle inhibitor P16; melanocortin 1 receptor (MC1R), which is responsible for synthesizing dark pigments triggered by UV light; cyclin-dependent kinase 4 (CDK4), a contributor to the regulation of cell cycle; Ras, which regulates cell division; and BRAF (v-Raf murine sarcoma viral oncogene homolog B1), which is involved in cell division and differentiation. In addition, defects in DNA repair mechanisms, as well as dysregulation of cellular growth and proliferation pathways are also associated with tumor progression in melanoma patients [23,24].

Genetic alterations in melanoma patients activate the RAS/RAF/MEK/ERK (MAPK) and the PI3K/PTEN/AKT (AKT) signaling pathways. Studies have shown that melanoma cell growth can be suppressed by simultaneously inhibiting ERK and PI3K signaling. The MAPK (mitogen-activated protein kinase) pathway can modulate downstream pathways of multiple receptors, including cytokine receptors, heterotrimeric G-protein-coupled receptors, and tyrosine kinase receptors. MAPK is a key pathway that is involved in melanoma and is activated by several mutation, such as NRAS and BRAF. The key downstream effectors of the MAPK pathway are BRAF and CRAF [23].

BRN2, a member of the POU domain family of transcription factors, plays an important role in melanoma progression and invasion. High levels of BRN2 expression may lead to higher invasive potential and also to inhibition of DNA repair and apoptosis in melanoma cell lines. In light of this data, it may be concluded that BRN2 is involved in many somatic mutations in melanoma patients [23].

MiRNAs are key contributors in the development and progression of melanoma, being deeply involved in many cellular processes, such as melanoma genesis, controlling of the cell cycle, tumor growth, proliferation, cell migration, invasion, resistance to therapy, and induction of apoptosis. Given this data, fundamental processes of melanoma cells can be affected by downregulating miRNAs [23].

Despite increasing interest in microRNAs (miRNAs) as diagnostic and prognostic biomarkers in melanoma, their variability according to patient sex and melanoma subtype remains poorly defined. While sex-related differences in miRNA regulation have been described in other cancers (breast, prostate, lung, colorectal, and liver), melanoma studies rarely stratify analyses by sex, and convincing evidence for sex-specific miRNA biomarkers is currently lacking. In contrast, emerging data indicate that miRNA expression profiles differ across melanoma subtypes, reflecting distinct genomic alterations and anatomical origins. These subtype-specific miRNA signatures may influence biomarker performance. Future studies integrating sex-based analyses with detailed genomic and anatomical classification are needed to refine miRNA-based stratification in melanoma [25].

Patients with early-stage melanoma have a mortality rate of approximately 11%, whereas those with advanced disease experience significantly higher rates. Once distant metastases develop, the median survival is around 6–9 months, with a 5-year survival rate of less than 5%. Consequently, there is a critical need to improve early-stage diagnosis and optimize treatment for advanced melanoma. In this context, understanding the molecular alterations underlying melanoma is essential for identifying novel therapeutic targets and developing biomarkers for diagnosis, treatment selection, and prognostic assessment [26].

## 4. MiRNA Biogenesis and Their Role in Oncogenesis

MiRNAs are key regulators of essential cellular processes and play a significant role in the development and progression of diseases, including skin cancers. By modulating gene expression, they influence protein production and can act as either oncogenes or tumor suppressors. MiRNAs control both the translation of proteins and the expression of genes. In cancer, these molecules can be secreted into blood, saliva, and urine, but they are also detectable in tissue samples. While circulating miRNAs are frequently analyzed in clinical studies, tissue-based miRNA analysis often yields more accurate and reliable results [1].

Primary miRNAs are transcribed by RNA polymerase II as long precursor transcripts, which are then cleaved in the nucleus by the RNase III endonuclease Drosha to generate a 60–70 nucleotide precursor miRNA (pre-miRNA). The pre-miRNA is further processed in the cytoplasm by another RNase III enzyme, Dicer, resulting in a miRNA–miRNA duplex. One strand of this duplex, the mature miRNA, is incorporated into the RNA-induced silencing complex (RISC), while the complementary strand is degraded. Binding of the miRNA-RISC to target mRNAs mediates gene silencing through translational repression or mRNA destabilization [7].

Thus, miRNAs are involved in numerous physiological and pathological processes and hold potential as biomarkers for tumor diagnosis and prognosis, as well as promising targets for therapeutic intervention [7].

Circulating miRNAs are found either within exosomes or bound to proteins such as Argonaute, reflecting distinct biological processes. Exosomal miRNAs are selectively packaged and often indicate active cellular signaling, whereas protein-bound miRNAs largely result from passive release. Distinguishing these forms is critical for sensitive and specific diagnostic and prognostic assays in melanoma and non-melanoma skin cancers. Exosome-enriched assays can enhance tumor specificity, while total circulating miRNA assays capture broader biological changes. Differences in miRNA profiles between cancer types and sexes may affect biomarker interpretation, underscoring the importance of considering sex as a variable. Pre-analytical factors, including sample type and processing, influence the recovery of exosomal versus protein-bound miRNAs. Understanding these nuances is essential for accurate interpretation and clinical application of circulating miRNA signatures [27,28].

MiRNAs play an important role in oncogenesis, due to the fact that dysregulation of miRNAs could interfere with the regulatory balance between tumor suppressors and oncogenes. The overexpression of several miRNAs with oncogenic roles (oncomiRs) promotes tumor development by downregulating suppressor genes, which can lead to increased proliferation and resistance to apoptosis [29]. In contrast, the loss of miRNAs with tumor-suppressive functions may promote oncogenic processes and malignant transformation [30]. MiRNA dysregulation can affect multiple cancer-related signaling pathways, including p53, RAS/MAPK, PI3K/AKT, and TGF-β, thereby influencing cell cycle control, DNA repair, cell migration, and angiogenesis [31]. Moreover, miRNAs play a key role in the regulation of epithelial–mesenchymal transition (EMT), a complex process of metastasis where some miRNAs inhibit EMT whilst others stimulate it [32]. At the same time, circulating miRNAs, transported in exosomes, have been proven to be useful biomarkers for diagnostic, prognostic, and treatment monitorization in many cancers [33]. MiRNAs play an important biological role, particularly in the development of experimental therapies based on miRNA mimics or antimiRs, which have been investigated in preclinical and clinical studies as potential anticancer treatments [30].

**Figure 1:**
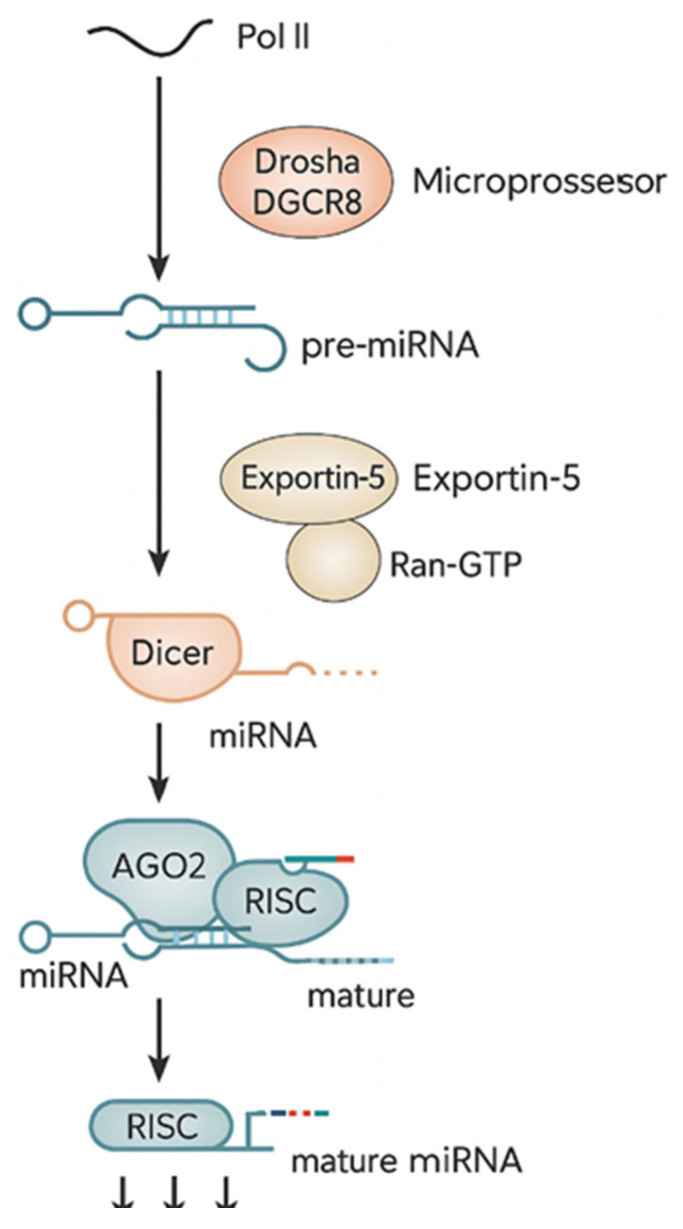
miRNA biogenesis.

The miRNA biogenesis is shown in Figure 1.

## 5. Main Technologies Used for the Identification of microRNAs from Biological Samples

Abnormal miRNA expression can be detected in tumor tissues as well as in circulating biological fluids. Recent studies indicate that miRNAs can enter the circulation either through encapsulation within extracellular vesicles or association with proteins such as Argonaute proteins (AGO) or high-density lipoproteins (HDLs) [3,5]. Circulating miRNAs may serve as indicators of underlying disease; however, their clinical use is limited by the complexity of the PCR-based detection methods commonly employed in laboratories [3].

Several technologies are available for the detection of miRNAs, each with distinct characteristics. Sequencing and microarray approaches allow for the analysis of many targets in a limited number of samples, whereas quantitative RT-PCR (qRT-PCR) is better suited for analyzing a few targets across a larger number of samples [3].

Quantitative PCR is widely used in cancer diagnostics to detect miRNAs from tissue or blood and includes methods such as RT-qPCR and TaqMan MicroRNA assays [3].

Microarray platforms are another commonly used technology, primarily for profiling known miRNA species. They offer the advantages of high-throughput analysis, rapid detection, and accurate quantification of hundreds of miRNA expression targets simultaneously. They were extensively studied in melanoma patients in order to identify miRNAs as diagnostic markers. Leidinger et al. were the first to discover 16 species of miRNAs from blood samples, which presented high sensitivity and specificity in order to differentiate between advanced melanoma cases (III and IV) and control subjects [34]. Later on, several studies reported similar results regarding miRNAs. Komina et al. identified over 1100 miRNAs species showing dysregulation in melanoma compared to melanocytic nevi [35]. Van Laar et al. in 2018 also discovered 38 miRNAs which can differentiate between melanoma and healthy subjects, using microarray platforms [36]. Despite their high sensitivity and specificity regarding miRNAs detection, microarray platforms are expensive and require qualified personnel, and for this reason, they are more adequate for miRNA detection in a specific disease [3].

MiRNA sequencing provides an alternative approach with distinct benefits over traditional methods like qPCR and microarrays. This technology is particularly useful during the discovery phase, where it helps in detecting a certain sequence of miRNA that is unknown. It can also offer a higher specificity within the same miRNA family. The most significant limitation of this technique is the computational system that supports data interpretation [3].

There are significant differences in the detection methods for unbound, protein-bound, and exosome-encapsulated miRNAs, reflecting their distinct biological properties and clinical relevance. Unbound miRNAs circulate freely in plasma or serum and can be extracted directly using standard RNA isolation methods, followed by qRT-PCR, digital PCR, or sequencing. These miRNAs are highly susceptible to RNase degradation and often reflect general tissue turnover rather than tumor-specific signals [27].

Protein-bound miRNAs, associated with Argonaute proteins or lipoproteins, are more stable and can be enriched using immunoprecipitation techniques targeting these protein complexes. While this increases assay sensitivity, protein-bound miRNAs may originate systemically, which can limit their tumor specificity [28].

In contrast, exosomal miRNAs are selectively packaged and secreted by tumor cells, providing enhanced stability and tumor-specific information. Detection requires prior exosome isolation using ultracentrifugation, size-exclusion chromatography, or immunoaffinity capture, followed by RNA extraction and quantification. Exosomal miRNAs have been shown to improve early diagnostic accuracy, prognostic stratification, and monitoring of therapy response in melanoma patients, making them the preferred fraction for clinical liquid biopsy assays [37].

Overall, the choice of miRNA fraction determines the sensitivity, specificity, and clinical utility of the assay: exosomal miRNAs provide the most tumor-specific signal, protein-bound miRNAs are stable and biologically informative, and unbound miRNAs are easier to detect but less tumor-specific.

**Figure 2:**
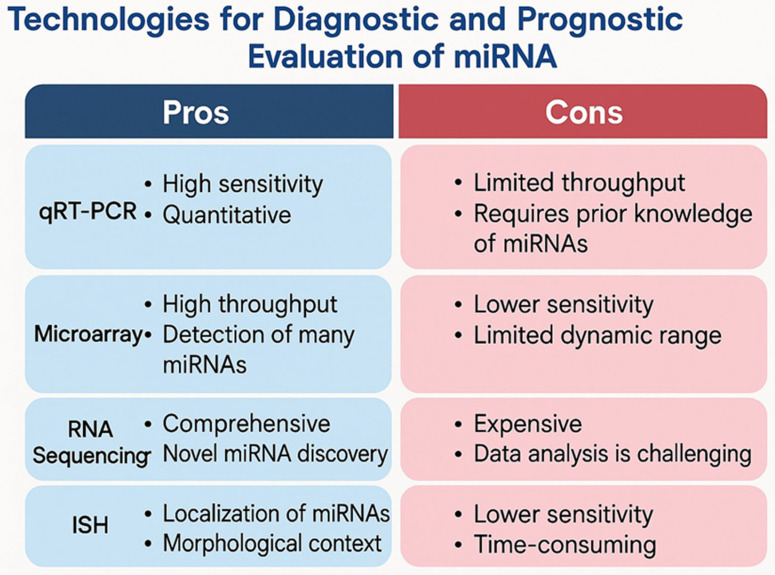
Technologies used for diagnostic and prognostic evaluation of miRNA.

Figure 2 shows the technologies used for diagnostic and prognostic evaluation of miRNA.

## 6. The Role of miRNAs in Basal Cell Carcinoma

Sand and colleagues made a significant contribution to understanding the role of miRNAs in basal cell carcinoma (BCC). They identified 16 miRNAs that were upregulated and 10 miRNAs that were downregulated in BCC compared to normal skin. The upregulated miRNAs included miR-17, miR-18a, miR-18b, miR-19b, miR-19b-1, miR-93, miR-106b, miR-125a-5p, miR-130a, miR-181c, miR-181d, miR-182, miR-455-3p, miR-455-5p, and miR-542-5p. The downregulated miRNAs were miR-29c, miR-139-5p, miR-140-3p, miR-145, miR-378, miR-572, miR-638, miR-2861, and miR-3196. The MAPK/ERK pathway plays a key role in cell proliferation through the activation of transcription factors that drive DNA synthesis, and mitotic progression MAPK/ERK-mediated phosphorylation of TRBP [HIV-1 transactivating response (TAR) RNA-binding protein] stabilizes the miRNA-processing complex, leading to globally increased miRNA levels, particularly through the upregulation of pro-growth miRNAs and the downregulation of growth-inhibitory miRNAs. MiR-17, miR-20a, and miR-92a are the pro-oncogenic miRNAs that are regulated by MAPK/ERK-induced TRBB phosphorylation. In this study, miR-17 was discovered to be significantly higher in BCC tissue compared to normal skin. The Hedgehog (HH) pathway, which is known to play a key role in BCC proliferation and survival, is also associated with miRNA regulation. As a downstream consequence of HH signaling, transforming growth factor-β induction leads to the downregulation of miR-141, miR-200a, miR-200b, miR-200c, miR-205, and miR-429. HH signaling additionally activates protein patched homolog 1 (PTCH1), the receptor for Sonic Hedgehog [38].

Based on these findings demonstrating upregulation of miR-17, miR-18a, miR-18b, and miR-19b-1 BCC, it has been shown that the miR-17-92 cluster cooperates with the Sonic Hedgehog pathway BCC [38].

In a subsequent study, Sand et al. reported that miR-143-5p and miR-145-5p are specifically downregulated in BCC compared to normal skin [39].

Pei Hu et al. reported that miR-34a expression is downregulated in BCC compared to healthy controls. In their study, serum samples from 86 BCC patients were analyzed and compared with those from 85 healthy individuals using real-time PCR. The results demonstrated that the serum levels of miR-34a were significantly lower in BCC patients than in the control group. Additionally, miR-34a expression correlated with tumor characteristics: larger tumors exhibited lower miR-34a levels compared to smaller tumors, and patients without lymph node metastasis had higher miR-34a expression than those with lymph node involvement. MiR-34a is involved also in other types of cancer, such as non-small cell lung cancer, where it can interact with other tumor suppressors, in order to induce arrest in the G1/S phase, downregulating the aberrant tumor proliferation. It also influences downstream apoptosis-related genes, like Bcl-2 and Survin, via signaling pathways such as Notch and c-met, in order to regulate tumor cell invasion and apoptosis. Consequently, it is believed that miR-34a is involved in the apoptosis of BCC cells, downregulating aberrant cell differentiation, proliferation, invasion, and migration [40].

Fastner et al. conducted a study to identify miRNAs capable of distinguishing BCC from normal skin, as well as differentiating between various BCC subtypes, including superficial, nodular/micronodular, and infiltrative forms. They identified 11 miRNAs with significant differential expression. Specifically, miR-383-5p and miR-145-5p distinguished all BCCs from normal skin. MiR-181c-5p, miR-181b-5p, and miR-95-3p showed differential expression between superficial BCC and other subtypes. miR-22-5p, miR-758-3p, and miR-30c-5p were able to specifically differentiate infiltrative BCC from nodular and micronodular subtypes [41].

Hui Sun et al. investigated the expressions of miR-451 in BCC compared to normal skin in 22 patients. Their study demonstrated that miR-451a levels in BCC tissue were significantly lower than in the corresponding healthy skin from the same individuals. The degree of downregulation varied among patients, ranging from approximately a twofold to over a tenfold decrease, highlighting a strong association between miR-451a expression and BCC. In order to validate these results, they investigate the expression of miRNA-451a in a BCC from a murine model, K5tTA/TREGLI1. It was discovered that high levels of miRNA-451a in tumor cells can inhibit cell growth via G1 cell cycle arrest. On the other hand, inhibiting miRNA-45 in primary cells stimulated cell growth and colony formation capacity. TBX1, which is a transcriptional factor implicated in cell proliferation and organ development, is the downstream target of miRNA-451a in BCC. Study shows an overexpression of TBX1 in BCC cells, being inversely correlated with miRNA-451a. Consequently, this study demonstrated a strong correlation between mi-RNA451a/TBX1 axis and BCC tumorigenesis [42].

Another study by X. Mi et al. investigated miR-18a expression in BCCs compared to normal skin and in A431 cells compared to HaCat cells. They reported a significant upregulation of miR-18a in BCC tissue and A431 cells. Downregulation of miR-18a can reduce proliferation in A431 cells [43].

Chang et al. explored the potential role of miR-197-5p as a candidate for further investigation into the mechanisms of BCC metastasis. Their findings indicated that miR-197-5p is expressed in both metastatic and non-metastatic BCC. Furthermore, fibroblast migration was significantly reduced by a synthetic inhibitor of miRNA-197, although it did not affect invasion [44]. The expression and functional roles of miRNA in BCC are presented in Table 1.

## 7. The Role of miRNAs in Cutaneous Squamous Cell Carcinoma

MiRNAs have been investigated as potential biomarkers and therapeutic targets due to their oncogenic and tumor-suppressive roles, with possible applications in the diagnosis and treatment of cutaneous squamous cell carcinoma (cSCC) [35]. Understanding the role of miRNAs in cSCC requires identifying the genes they regulate. Recent advances have significantly improved our knowledge of miRNA-regulated genes and their functional roles [45,46].

A 2017 study reported that miR-31 expression is elevated in cSCC. Specifically, miR-31 levels were significantly higher in the cSCC cell line A-431 compared to HaCaT cells. Previous studies have reported similar findings. Wang A et al. [47] investigated miRNA dysregulation in cSCC and observed a marked increase in miR-31 expression in cSCC tissue samples relative to normal skin. To validate these results, they compared cSCC tissues with both healthy skin and actinic keratoses (AKs). Using RT-PCR on samples from healthy individuals, AK lesions, and cSCC tissues, they found that miR-31 was not upregulated in AK compared to normal skin but was significantly elevated in cSCC relative to both healthy skin and AK. These findings suggest that miR-31 is specifically upregulated in invasive lesions and may represent a potential therapeutic target [47,48,49]. With the aim of discovering the molecular mechanism of miR-31, a bioinformatic analysis was performed. It was discovered that RhoBTB1 presented two binding sites for miR-31. In order to demonstrate that RhoBTB1 was directly targeted by miR-31, a luciferase reporter gene assay was conducted. Results showed that miR-31 overexpression decreased RhoBTB1 expression, whilst inhibition of miR-31 increased its levels, confirmed also by Western Blot analysis. These findings indicate that RhoBTB1 is a direct target of miR-31 and suggest that miR-31 upregulation in cSCC contributes to reduced RhoBTB1 expression [48].

Canueto J et al. investigated the expression of miR-205 and miR-203 in patients with cSCC. They found that miR-205 was significantly upregulated in infiltrative and poorly differentiated cSCC, which are generally associated with invasion, perineural involvement, and poor prognosis. In contrast, miR-203 was primarily expressed in well-differentiated tumor areas and was not associated with tumors exhibiting aggressive or poor-prognosis features. It has been demonstrated that miR-203 inhibits P63, resulting in cell differentiation and suppression of stem cell characteristic, whilst miR-205 inhibits E-CADHERIN and leads to expansion of stem cell compartment. Assessing the correlation between miR-203 and miR-205 with P63 and E-CADHERIN as epithelial differentiation markers, investigators discovered that, as they expected, P63 was correlated with poorly differentiated tumors, being overexpressed in undifferentiated areas. On the other hand, E-CADHERIN is expressed in well-differentiated areas and uncommon in undifferentiated areas [50].

Gong Z.H. et al. examined the potential role of miR-221 in cSCC. miR-221 is part of the miR-221/222 cluster located on the X chromosome. This study demonstrated that miR-221 expression was significantly increased in cSCC tissue samples, suggesting that miR-221 may represent a promising biomarker and potential therapeutic target in patients with squamous cell carcinoma. In order to determine the exact roles of miR-221 in cSCC cell lines, investigators assessed the cell proliferation by MTT assay following transfection with miR-221 mimics or inhibitors. The results showed that miR-221 upregulation significantly enhanced cell proliferation, whereas miR-221 downregulation markedly suppressed proliferative activity. Furthermore, colony formation assays demonstrated that cells transfected with miR-221 mimics generated a greater number of colonies compared with control cells, while the opposite effect was observed in cells transfected with the miR-221 inhibitor [51].

In another study, Zhang et al. found that miR-20a was markedly downregulated in cSCC tissue compared with normal skin, suggesting a possible tumor-suppressive role of miR-20a in cSCC. Following relative expression analyses, investigators examined the association between miR-20a and clinicopathological characteristics. The study showed a significant correlation between miR-20a expression and the TNM stage of cSCC. Low levels of miR-20a were more frequently observed in tumor at advanced TNM stages. These findings suggest that aberrant miR-20a expression may play a critical role in the tumorigenesis and progression of cSCC [52].

Chen et al. reported that miR-346 expression was elevated in cSCC tissues relative to normal skin, while SRCIN1 expression was reduced. Notably, their analysis showed a strong inverse correlation between miR-346 and SRCIN1 levels in cSCC samples [53]. The expression and functional roles of miRNA in SCC are presented in Table 2.

## 8. The Role of miRNA in Merkel Cell Carcinoma

Merkel cell carcinoma (MCC) is a rare but highly aggressive form of non-melanoma skin cancer, typically associated with an unfavorable prognosis. It originates in the basal layer of the epidermis and most often develops on sun-exposed areas of the skin. MCC has also been linked to infection with the Merkel cell polyomavirus (MCPyV) [7,54].

Ning et al. reported that eight miRNAs (miR-502-3p, miR-9, miR-7, miR-340, miR-182, miR-190b, miR-873, and miR-183) were upregulated, while three miRNAs (miR-3170, miR-125b, and miR-374c) were downregulated in MCC tissue samples, with miR-182 showing particularly high expression in malignant cells. In MCPyV-negative MCC cell lines, they observed a decrease in the expression of four miRNAs (miR-182, miR-183, miR-190b, and miR-340). Based on these findings, the authors suggested that these miRNAs may serve as potential diagnostic markers in MCPyV-positive MCC [7,55].

Fan K et al. showed that the tumor-suppressor gene Atonal homolog 1 (ATOH1) regulates miR-375 by binding to and enhancing its activation. MiR-375 is the most abundant microRNA in MCC. Experimental models demonstrated that loss of ATOH1 in MCC cell lines leads to reduced expression of miR-375. Interestingly, overexpression of ATOH1 has been linked to a higher metastatic risk. Additionally, MCPyV infection appears to promote carcinogenesis partly by stimulating ATOH1 activity [7,56].

A notable finding was reported by Konstantinell et al. regarding the clinical value of circulating miRNAs as potential biomarkers for MCC. Using RT-PCR, the authors analyzed exosomal miRNAs and detected several candidates, including miR-30a, miR-125b, miR-183, miR-190b, and miR-375 [21].

## 9. The Role of miRNA in Melanoma

To date, there is no biomarker with sufficient specificity or sensitivity for the early detection or prognosis of melanoma. Although some studies have reported potential associations between certain molecules and melanoma, the available evidence is still not enough. An ideal biomarker should be highly specific for the disease and sensitive enough to minimize false-positive or false-negative results. Early detection of melanoma—preferably before clinical symptoms appear—is crucial, as it would allow correlation of biomarker levels with disease progression or therapeutic response. In addition, such a biomarker should be easily accessible, measurable in body fluids, and clinically applicable [5].

With regard to microRNAs as blood-based biomarkers for melanoma, Fogli et al. reported elevated levels of miR-150-5p, miR-149-3p, and miR-15b-5p and reduced levels of miR-193a-3p, and miR-524-5p in melanoma patients [5,57]. Additionally, the expression of miR-134-5p and miR-320a-3p was found to be downregulated in plasma samples from patients with stage 0 melanoma compared to healthy controls. These circulating miRNAs have been proposed as potential diagnostic biomarkers, with a specificity greater than 90% and a sensitivity exceeding 80% [58].

Babapoor et al. examined fixed tissue samples and found that miR-211 expression was elevated in common and atypical nevi but reduced in melanoma [59].

Barbato et al. demonstrated that miR-181a/b is involved in drug resistance in melanoma. By comparing gene expression in melanoma cell lines that were either sensitive or resistant to the BRAF inhibitor dabrafenib, they observed that miR-181a/b levels were higher in BRAFi-sensitive cells. Moreover, inhibition of miR-181a/b restored sensitivity to BRAFi in resistant melanoma cells. Their results also showed that miR-181a/b promoted resistance to BRAFi and suppressed tumor growth in patient samples with BRAF V600 mutations. Overall, miR-181a and miR-181b show potential as future biomarkers in melanoma [60].

Zhou et al. investigated miR-128-3p and identified it as a tumor suppressor in melanoma. They found that miR-128-3p levels were reduced in melanoma cells and that its upregulation inhibited melanoma cell proliferation and migration while promoting apoptosis [61].

Similarly, miR-18a-5p has been implicated in melanoma progression and pathogenesis. Guo Y et al. analyzed tissue samples from 20 patients and found that miR-18a-5p was upregulated in melanoma, suggesting a pro-oncogenic role. Moreover, inhibition of miR-18a-5p significantly reduced melanoma cell proliferation compared to normal cells [62].

MiR-221 was among the first microRNAs identified in melanoma and remains one of the most extensively studied in the literature [63,64]. Notably, as melanoma progresses, the expression of the receptor tyrosine kinase c-KIT decreases, while levels of miR-221/222 increase, indicating a negative correlation between miR-221/222 and c-KIT expression [54,56]. Studies have shown that miR-221 is abnormally expressed in melanoma cells and can also be detected in the circulating fluids of melanoma patients. Its expression has been associated with tumor thickness, disease progression, and late recurrence, suggesting that miR-221 may serve as a valuable biomarker for melanoma [26,63,64,65].

MiR-146a is among the most extensively studied microRNAs in melanoma [26]. Akseneko M et al. reported that both miR-146a and miR-146b are upregulated in melanoma patients compared to healthy controls [66].

In addition, five other microRNAs—miR-149-3p, miR-193a-3p, miR-150-5p, miR-155-5p, and miR-21-5p—have been identified as potential biomarkers. MiR-149-3p plays a key role in cell migration [67]. MiR-193a-3p is associated with the presence of BRAF mutations in melanoma [44]. MiR-150-5p is involved in hematopoiesis and the regulation of genes critical for stem cell differentiation [68,69]. MiR-155-5p participates in both normal and pathological cellular pathways [63,70]. MiR-21-5p is linked to several cancer-related genes, including PTEN, TIMP3, RHOB, COAD, PDCD4, and BTG2 and is implicated in multiple pathological mechanisms, including oncogenesis. It has also been found to be upregulated in plasma and other bodily fluids [71]. The expression and functional roles of miRNA in melanoma are presented in Table 3.

## 10. Dysregulation Across Models and Patient Data in Skin Cancer

Cell-line models reliably reproduce the direction of miRNA upregulation or downregulation observed in skin cancers and are therefore useful for identifying candidate diagnostic biomarkers. However, they fail to capture the complexity of the tumor microenvironment and systemic influences, which limits their prognostic relevance. Animal models provide a more physiologically relevant context by incorporating tumor–host interactions and systemic release of circulating miRNAs, resulting in improved concordance with patient data, particularly for diagnostic applications. Nevertheless, species-specific differences constrain their ability to fully model long-term clinical outcomes. Human tumor tissues remain the gold standard for validating the biological relevance of miRNA dysregulation, as they directly reflect disease stage and tumor heterogeneity. In contrast, miRNAs detected in human biofluids offer strong potential for non-invasive diagnostics and prognostics, but their interpretation is influenced by pre-analytical variables and by whether miRNAs are exosome-associated or protein-bound. Together, these observations highlight the necessity of validating miRNA biomarkers across multiple experimental systems before clinical translation [27,28,29]. The concordance of miRNA dysregulation across models and patient data in skin cancers are shown in Table 4.

## 11. Discussion

Despite advancements in prevention, diagnostic techniques, and therapeutic strategies, the molecular mechanisms underlying both melanoma and non-melanoma skin cancers (NMSCs) remain incompletely elucidated. Increasing evidence highlights the pivotal role of microRNAs (miRNAs) as post-transcriptional gene regulators influencing key cancer-related pathways, including cellular proliferation, differentiation, apoptosis, invasion, and metastasis. Depending on their molecular targets, miRNAs may function as oncogenes or tumor suppressors. The present synthesis of available data further underscores the relevance of miRNA dysregulation across distinct cutaneous malignancies.

Basal cell carcinoma (BCC), the most common NMSC, demonstrates distinct miRNA expression signatures that correlate with histopathological subtypes and tumor behavior. Several miRNAs—including miR-383-5p, miR-145-5p, and miR-18a—effectively distinguish BCC tissue from normal skin, suggesting utility as diagnostic markers [41,43]. Notably, miR-34a is consistently downregulated in BCC and is associated with larger tumor diameter and lymph node involvement, supporting a tumor-suppressive role [40].

Subtype-specific patterns have also been reported. Superficial BCC exhibits upregulation of miR-181c-5p, miR-181b-5p, and miR-95-3p, whereas infiltrative BCC demonstrates increased levels of miR-22-5p, miR-18a-3p, and miR-708-5p. Additional molecules, including miR-758-3p and miR-30c-5p, are upregulated in infiltrative compared with nodular or micronodular BCC [38]. The involvement of miR-197-5p in invasion and metastasis further supports the contribution of miRNAs to tumor aggressiveness [44].

Cutaneous squamous cell carcinoma (cSCC) also displays miRNA alterations linked to tumor progression. miR-31, one of the most extensively studied molecules in cSCC, is markedly upregulated compared with both normal skin and actinic keratoses, suggesting a role in the transition from premalignant to invasive lesions [46,47,48]. High expression of miR-205 is associated with poor prognosis and invasive tumors, whereas miR-203 appears reduced in aggressive cSCC and is linked to more favorable outcomes [50]. miR-221, upregulated in cSCC, may further contribute to discrimination between malignant and non-malignant skin [51].

Although less common, Merkel cell carcinoma (MCC) demonstrates distinct miRNA dysregulation. miR-375 is the predominant species involved and is strongly associated with the tumor-suppressor gene ATOH1, suggesting a role in tumor differentiation and progression. However, additional studies are required to validate its function and clinical relevance [7,21].

Melanoma exhibits widespread miRNA alterations, highlighting its intricate molecular landscape. Among the upregulated miRNAs, miR-18a-5p promotes oncogenesis and disease progression, whereas miR-128-3p is reduced and appears to function as a tumor suppressor [58,59]. Upregulation of miR-146a is consistently observed in melanoma relative to healthy skin, further implicating it in tumorigenesis [26].

Additional miRNAs, including miR-149-3p, miR-150-5p, and miR-155-5p, influence cell differentiation and migration, while miR-193-3p correlates with BRAF mutation status. Drug resistance represents an emerging challenge in melanoma therapy, and the association of miR-181a/b with treatment resistance highlights a potential biomarker for predicting therapeutic response [57,63,67,68,69].

The diagnostic and prognostic values of miRNAs in skin cancers have been evaluated both as individual markers and multi-miRNA panels, with patterns varying by cancer type. In melanoma, individual miRNAs such as miR-21 and miR-221/222 are upregulated and correlate with tumor progression and poor prognosis, while miR-125b and miR-205 are downregulated in aggressive tumors. Multi-miRNA panels, often including these miRNAs, improve diagnostic specificity and prognostic accuracy, particularly when derived from circulating or exosomal miRNAs [14,37,73].

In non-melanoma skin cancers (BCC and SCC), upregulation of miR-21 and downregulation of miR-203 are frequently observed. Combining multiple miRNAs modestly enhances diagnostic performance, although research on prognostic panels is more limited [14,73]. Overall, multi-miRNA signatures provide superior sensitivity and specificity compared to individual miRNAs, especially in melanoma.

Several miRNAs are dysregulated in both non-melanoma skin cancers (NMSC), including basal cell carcinoma (BCC) and cutaneous squamous cell carcinoma (cSCC), and melanoma, indicating partial overlap in molecular pathways underlying skin carcinogenesis. For example, miR-21 and miR-221/222 are upregulated in both melanoma and NMSC, promoting proliferation, invasion, and resistance to apoptosis, while miR-203 and miR-34a often act as tumor suppressors and are downregulated in multiple skin cancer types [1,3,5,7,14]. Similarly, miR-31 is frequently overexpressed in cSCC but has also been implicated in melanoma progression, reflecting partially shared oncogenic mechanisms [2,48].

Despite these overlaps, the expression levels and patterns are often cancer-type specific, allowing potential differentiation. Clinicians can integrate miRNA profiling with histological and immunohistochemical analysis to accurately distinguish between melanoma and NMSC. For instance, the combination of tumor-specific miRNA signatures with tissue morphology enables identification of melanoma subtypes versus BCC or cSCC, particularly when histology alone is ambiguous [5,6,14]. Emerging liquid biopsy approaches measuring circulating or exosomal miRNAs further support non-invasive differentiation, although tissue confirmation remains the gold standard.

Overall, overlapping miRNAs highlight shared oncogenic pathways, but the relative expression patterns, when combined with histological analysis, allow clinicians to distinguish between melanoma and non-melanoma skin cancers reliably. Incorporating miRNA profiles into diagnostic workflows may enhance accuracy, particularly in challenging cases or when minimally invasive sampling is preferred.

The clinical applicability of miRNAs is strengthened by their detectability in both tissue biopsies and body fluids. Tissue-derived miRNAs provide high analytical sensitivity but depend on adequate biopsy sampling. Conversely, circulating miRNAs offer a non-invasive alternative suitable for longitudinal monitoring, although variability in sensitivity remains a limitation. Liquid biopsy, in combination with dermoscopic evaluation, may enhance risk stratification and improve early detection of aggressive skin cancers [74,75,76].

Multiple technologies—such as microarray profiling, quantitative PCR, RT-qPCR (the current diagnostic gold standard), next-generation sequencing, miRNA enzyme immunoassays, and multiplexed assays—enable sensitive and high-throughput miRNA quantification. Their continued refinement will be essential for translating miRNA research into clinical practice [77,78,79].

## 12. Conclusions

Overall, current findings reinforce the considerable potential of miRNAs as diagnostic, prognostic, and predictive biomarkers in both melanoma and NMSCs. Their subtype specificity, association with tumor aggressiveness, and involvement in treatment resistance highlight their value for precision oncology. However, the heterogeneity of existing studies and methodological variability underscore the need for standardized protocols and validation in large, well-characterized patient cohorts.

A more comprehensive understanding of miRNA regulatory networks may ultimately contribute to improved diagnostic accuracy, individualized prognostication, and the development of novel therapeutic strategies, thereby enhancing clinical outcomes for patients with cutaneous malignancies.

## Figures and Tables

**Table 1:** Expression and functional roles of miRNA in BCC.

Ref	Expression	miRNA	Role
Sand M. et al. [39]	upregulated	miR-17	pro-growth miRNA regulated in vitro by MAPK/ERK-induced phosphorylation of TRBP
		miR-18a, miR-18b	cell proliferation and the suppression of apoptosis
		miR-19b, miR-19b-1	enhanced cell proliferation and the suppression of apoptosis
		miR-93	transcription factor E2F1 (E2 promoter binding factor 1) target gene
		miR-106b	transcription factor E2F1 is a target gene
		miR-125a-5p	induces apoptosis
		miR-130a	regulatory effect on the apoptosis
		miR-181c, hsa-miR-181c*, 181d	targets NOTCH4 (neurogenic locus Notch homolog-4) and KRAS (Kirsten rat sarcoma virus)
		miR-182	negatively regulates human Forkheadbox O1 (FOXO1)
		miR-455-3p, miR-455-5p miR-542-5p	not mentioned
	downregulated	mir-143-5p mir-145-5p	targets EGFR
Pei Hu et al. [40]	downregulated	mir-34a	regulates the apoptosis of cells, and inhibits abnormal cell differentiation, proliferation, invasion, and migration
Fastner et al. [41]	downregulated	Mir-383-5p, miR-145-5p	discriminates between BCC subtypes and normal skin
Hui Sun et al. [42]	downregulated	miRNA-451a	suppresses cell growth through G1 cell cycle arrest
X Mi et al. [43]	upregulated	mir-18a	discriminates between BCC tissues and normal skin; an oncogenic role through a novel Akt/mTOR/Beclin 1/LC3 axis and the antitumor effects of miR-18a inhibitor may make it suitable for BCC therapy
Chang et al. [44]	upregulated	miR197-5p	potential role in metastasis process

**Table 2:** Expression and functional roles of miRNA in SCC.

Ref	Expression	miRNA	Role
Wang A et al. [47]	upregulated	miR-31	promotes migration and invasion of cSCC cells
Canueto J et al. [50]	upregulated	miRNA-205	maintains a poorly differentiated and more aggressive epithelial phenotype in the tumors
		miRNA-203	tumor suppressor in human cSCC
Gong ZH et al. [51]	upregulated	miR-221	promotes cell proliferation
Zhang et al. [52]	downregulated	mir-20a	associates with the TNM stage of cSCC
Chen et al. [53]	upregulated	mir-346	promotes cSCC cell proliferation and migration by directly targeting SRCIN1

**Table 3:** Expression and functional roles of miRNA in melanoma.

Ref	Expression	MiRNA	Role
Fogli et al. [57]	upregulated	miR-150-5p	increases tumor immunoresistance by post-transcriptionally downregulating perforin-1 in mouse NK cells
		miR-149-3p	p53-dependent survival by increasing the expression of the anti-apoptotic Mcl-1 protein
		miR-15b-5p	increasing cell proliferation and decreasing apoptosis in melanoma cell lines
	downregulated	Mir-193a-3p	tumor suppressor in many human cancers, including melanoma
		miR-524-59	inhibits melanoma cell proliferation and migration in human melanoma cell lines
Babapoor et al. [59]	downregulated	miR-211	potent tumor suppressor—influencing gene pathways involved in cell invasion
Barbato et al. [60]	upregulated	miR-181a/b	new potential modulators of melanoma resistance to BRAF inhibitors; miR-181a/b depletion may trigger tumor growth in vitro
Zhou et al. [61]	downregulated	miR-128-3p	tumor suppressor
Guo Y et al. [62]	upregulated	miR-18a-5p	inhibition of EPHA7 expression induces melanoma cell proliferation and suppresses apoptosis and autophagy
	upregulated	miR-221	loss of expression of the tyrosine kinase receptor c-KIT
Aksenenko et al. [66]	upregulated	miR-146a	not mentioned
	upregulated	miR-149-3p miR-193-3p miR-150-5p miR-155-5p mir-21-5p	not mentioned
Sole C: et al. [58]	downregulated	MiR-134-5p	tumor suppressor, regulates cell proliferation, apoptosis, invasion, and migration
		MiR-320a-3p	inhibitor of cell proliferation

**Table 4:** Concordance of miRNA dysregulation across models and patient data in skin cancers.

Data/Model Type	What miRNA Up-/Downregulation Reflects	Concordance With Patient Data	Utility (Diagnostic/Prognostic)	Key References
Cell Lines	Intracellular miRNA expression; direct molecular mechanisms (proliferation, apoptosis, invasion)	Moderate—directionality often conserved, magnitude differs	Diagnostic: High (screening); Prognostic: Low	Wang et al., 2016 [27]; Hayes et al., 2014 [72]
Animal Models	Tumor tissue expression plus systemic release of miRNAs (including circulating miRNAs)	Moderate–High—closer to human context than cell lines	Diagnostic: Moderate–High; Prognostic: Moderate	Peng & Croce, 2016 [29]; Wang et al., 2016 [27]
Human Tumor Tissue	Tumor-specific miRNA profiles reflecting disease stage and progression	Reference standard for biological validation	Diagnostic: High; Prognostic: Moderate	Hayes et al., 2014 [72]; Peng & Croce, 2016 [29]
Human Biofluids (Serum/Plasma)	Circulating miRNAs (exosomal and protein-bound	Variable, influenced by biological and pre-analytical factors	Diagnostic: Moderate–High; Prognostic: Moderate	Nik Mohamed Kamal & Shahidan, 2020 [28]; Wang et al., 2016 [27]

## Data Availability

The original contributions presented in the study are included in this article; further inquiries can be directed to the corresponding author.

## Abbreviations

The following abbreviations are used in this manuscript:

BCC	basal cell carcinoma
cSCC	cutaneous squamous cell carcinoma
MCC	Merkel cell carcinoma
BD	Bowen’s disease
AK	actinic keratosis
miRNAs	microRNAs
pri-miRNAs	primary miRNAs
DNA	deoxyribonucleic acid
pre-miRNAs	precursor miRNAs
EMT	epithelial-to-mesenchymal transition
NMSC	non-melanoma skin cancers
MSC	melanoma skin cancer
UV	ultraviolet
SCC	squamous cell carcinoma
CDKN2A	cyclin-dependent kinase inhibitor 2A
MC1R	melanocortin 1 receptor
CDK4	cyclin-dependent kinase 4
BRAF	v-Raf murine sarcoma viral oncogene homolog B1
AGO	Argonaute proteins
HDL	high-density lipoprotein
qRT-PCR	quantitative RT-PCR
E2F1	E2 promoter binding factor 1
NOTCH4	neurogenic locus Notch homolog-4
KRAS	Kirsten rat sarcoma virus
MAPK	mitogen-activated protein kinase
TRBP	TAR-RNA binding protein
EGFR	Epidermal Growth Factor Receptor
MCPyV	Merkel cell polyomavirus
ATOH1	Atonal homolog 1
PTEN	Phosphatase and Tensin Homolog
TIMP3	Tissue Inhibitor of Metalloproteinases 3
RHOB	Ras Homolog Family Member B
COAD	colon adenocarcinoma
PDCD4	Programmed Cell Death Protein 4
BTG2	BTG Anti-Proliferation Factor 2
NK cells	natural killer cells
EPHA7	EPH Receptor A7
